# *Ex Vivo* Fluorescence Molecular Tomography of the Spine

**DOI:** 10.1155/2012/942326

**Published:** 2012-11-08

**Authors:** Monish Pimpalkhare, Jin Chen, Vivek Venugopal, Xavier Intes

**Affiliations:** Department of Biomedical Engineering, Rensselaer Polytechnic Institute, Troy, NY 12180-3590, USA

## Abstract

We investigated the potential of fluorescence molecular tomography to image *ex vivo* samples collected from a large animal model, in this case, a dog spine. Wide-field time-gated fluorescence tomography was employed to assess the impact of multiview acquisition, data type, and intrinsic optical properties on the localization and quantification accuracy in imaging a fluorescent inclusion in the intervertebral disk. As expected, the TG data sets, when combining early and late gates, provide significantly better performances than the CW data sets in terms of localization and quantification. Moreover, the use of multiview imaging protocols led to more accurate localization. Additionally, the incorporation of the heterogeneous nature of the tissue in the model to compute the Jacobians led to improved imaging performances. This preliminary imaging study provides a proof of concept of the feasibility of quantitatively imaging complex *ex vivo* samples nondestructively and with short acquisition times. This work is the first step towards employing optical molecular imaging of the spine to detect and characterize disc degeneration based on targeted fluorescent probes.

## 1. Introduction

Fluorescence molecular tomography (FMT) is an emerging preclinical imaging modality that provides means to retrieve the 3D bio-distribution of a fluorescent marker noninvasively [1, 2]. FMT is a subsurface imaging modality that involves capturing 2D surface measurements and casting an inverse problem to obtain quantitative 3D maps. FMT is based on a forward model of light excitation propagation in diffusive medium and light emission propagation from fluorophores to the detectors on the boundary. Localization and quantification of pathological tissue can be obtained by retrieving the spatially varying fluorophore distribution from the measurements obtained from the excitation and emission wavelengths.

One key aspect of FMT is the use of near-infrared (NIR) fluorescence probes, which have been identified to be the most effective for *in vivo* imaging. In this spectral window, the attenuation of biotissues is minimal, allowing the use of low laser power sources, and hence, no tissue damage even under prolonged illumination. Also, the relatively low absorption properties of the samples at these wavelengths allow to sense minute concentration of the fluorophore. FMT typically offers higher sensitivity compared to other modalities, such as micro CT or micro MRI [3] and is relatively inexpensive. FMT is also capable of imaging various molecular targets using multiple agents with different spectral or lifetime characteristics [4–6]. 

Even though the NIR window exhibits the lowest light attenuation overall, the use of FMT for preclinical studies has been limited to small animals, especially murine models. Use of FMT for larger animal models is precluded due to relative high-attenuation of the tissue and larger volumes to be imaged compared to mice. However, while mice provide proof of principle and allow testing of a variety of therapeutic modalities, mouse models have some limitations, as only short-term experiments can be performed, their homogenous genetic background is unlike humans, and the knockout models do not always faithfully represent the human disease. Naturally occurring large animal models of human diseases have become increasingly important despite the costs and the extensive clinical attention they require because of their similarities to human patients. Large animals are reasonably outbred, are more similar in size to a neonate or small child providing an opportunity to address issues related to scaling up therapy, and many physiological parameters including the immune system are more similar to those in humans versus those in mice. The list of large animal models of human diseases has become quite long and continues to increase [7]. For such animal models, however, it is unlikely that whole-body FMT in transmission will ever be achieved though FMT can still play a critical role in molecular imaging studies of these preclinical models by offering the opportunity to image nondestructively *ex vivo* large harvested samples.

Optical imaging of *ex vivo* samples is a staple technique in biomedical research. For thick specimen (>~1 mm), the main technique employed is histological sectioning of the sample to obtain high-resolution spatial information. However, this approach is destructive and yields incomplete 3D rendering. So far, it is not possible to noninvasively image non-processed specimen at microscopic resolution because light is highly scattered in such relatively thick tissues. To overcome this obstacle, tissue clearing procedures have been devised that minimize scattering and hence, render the specimen optically transparent [10]. For instance, Ertürk et al. proposed a new tissue clearing procedure that allows achieving cellular resolution imaging of axon up to 4 mm deep in an unsectioned spinal cord of adult GFP-M mice [11]. Moreover, millimeter scale mesoscopic imaging techniques such as selective plane illumination microscopy [12] and optical projection tomography [13] have been developed recently and applied successfully to image millimeter thick cleared samples. These techniques provide unique tools for applications, such as developmental biology. They also benefit from the use of clearing agent to image thick specimen at the cost of a reduced resolution. However, such procedure requires dehydratation of the specimen and the use of a lipid-dissolving (THF) agent (in the case of spine imaging as in [11]). Hence, it cannot be employed to image soft tissue (such as the intervertebral disk in the spine). Furthermore, these techniques are not adequate for larger sample (>~5 mm). Then, the scattering is becoming predominant and to image specimen at this scale, it is required to employ mesoscopic or macroscopic techniques that harness scattered photons, such as FMT [14] (FMT and OPT are based on the same principles, but FMT light modeling includes multiple-scattered photons).

Herein, we describe the first attempt towards FMT of the *ex vivo* dog spine in its entirety (~2 cm thick). More precisely, we focus on imaging of the intervertebral disk (soft tissue) with the long-term goal to apply this new method in orthopedic research to investigate disk degeneration's role in low back pain. To establish the feasibility and performances of FMT in this challenging scenario, an *in silico* study is performed based on a synthetic spine model derived from an experimental specimen. The *in silico* study investigates the effect of multiview, data type, and optical heterogeneous background on the localization and quantification of a fluorescent inclusion in the intervertebral disk. Furthermore, proof of principle of the methodology is performed experimentally.

## 2. Materials and Methods 

### 2.1. Large Animal Spine Model

**Figure 1:**
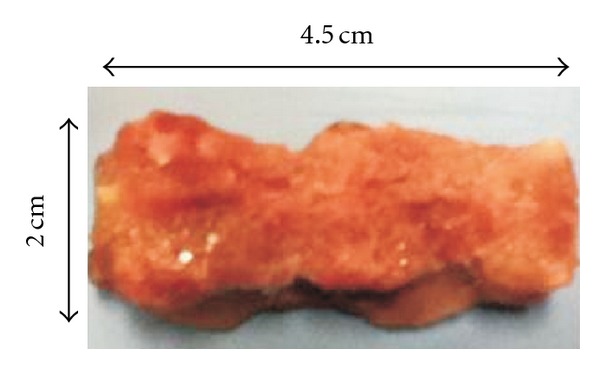
Dog spine specimen which was used in this study. The spine has been sectioned to be made as uniform as possible. This particular specimen contains 2 intervertebral discs and 3 vertebrae. The specimen was reduced to 2 vertebrae and 1 disc for the imaging study. The overall dimensions of the specimen are approximately 4.5 cm by 2 cm by 2 cm.

Models of disc degeneration are mainly used to address scientific questions relating to disease progression and treatment [15]. Many studies in pre-clinical settings on disc degeneration have been done on species, such as rabbits and dogs, due to the comparative ease of access versus human tissue. Rabbit and dog spines have been used in diagnostic radiology and ultrasound studies to evaluate the performance of these modalities and to further interpret their results. Herein, we focus on the first foray into establishing the potential of fluorescence optical tomography for imaging the intervertebral disc using a well-established dog spine model.  Figure 1 shows the dog spine specimen used in this study. The specimen contains 2 intervertebral discs and 3 vertebrae. The processes, muscles, and soft tissue around the vertebrae have been removed to give it an approximately cylindrical geometry.

To mimic the uptake of a targeted fluorescent molecular probe, a fluorophore was injected directly into the intervertebral disc of the spine using a 27 g needle. The fluorophore used in this study was Indocyanine Green (ICG), which is an FDA approved compound [16]. To create the fluorophore solution, 2.5 nmol of ICG was mixed with 180 *μ*L of ethanol. To achieve accurate localization of the fluorophore into the disk, a negative pressure was created inside the disc by using an empty syringe from one end and by injecting the fluorophore with another syringe located on the opposite side of the disk. This approach allowed obtaining a localized distribution of the fluorophore within the disk.

### 2.2. Wide-Field Time Resolved FMT

**Figure 2:**
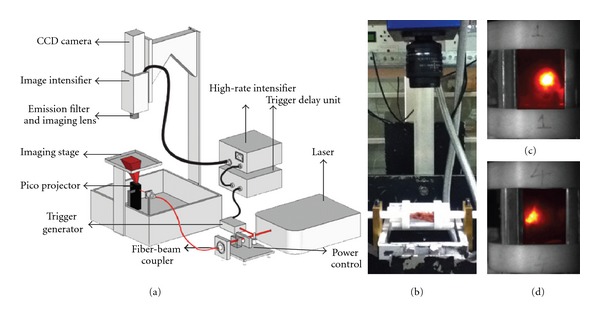
(a) Wide-field time-resolved optical imaging system; (b) *ex vivo* spine positioned on the imaging platform within its holder; (c)-(d) typical noncontact fluorescence measurement in transmission of the spine for two views 180° apart (labeled 1 and 4).

To perform FMT of the *ex vivo* specimen, we employed a wide-field time-resolved FMT system developed at the Rensselaer Polytechnic Institute (RPI). The system is described in details in [17]. In brief, this original system is based on an innovative time-resolved wide-field illumination strategy that allows acquiring functional and fluorescence tomographic data sets at an unprecedented acquisition speed with high-spatial, temporal and potentially high-spectral density [18].  Figure 2(a) provides a schematic of the preclinical imaging platform. The system uses a tunable Ti:Sapphire laser (Mai Tai HP, Spectra-Physics, CA, USA), capable of generating 100 fs pulses at 80 Mhz and at *λ* = 780 nm. A pico projector is used to generate wide-field illumination patterns with the potential to spatially code light over an 8 cm × 4 cm area with 256 levels of gray. The transmitted light is detected by a gated CCD (PicoStar HR, LaVision GmbH, Germany) with a resolution of 1376 by 1040 pixels. A 32 nm band pass filter centered at 832 nm (Semrock, USA) was used to collect the fluorescence emission.

### 2.3. Multimodal Multi-View Holder

Our wide-field fluorescence optical imaging system has been designed to image live murine models in the prone position. This configuration allows observing the animal under a physiologically non-stressing position. However, this approach is not optimal to capture full tomographic information as it allows acquiring only one view in transmittance. A number of studies have been done to develop multi-view FMT [19, 20]. Most of these studies though have been performed taking data with high angular sampling. Although complete 360 degree imaging gives excellent tomographic information, the experimental acquisition time is prohibitive and numerous studies are limited to single slice imaging, not full 3D tomography [21]. Herein, we developed a specimen holder to rotate the spine in intervals of 90 degrees for a total of 4 acquisition views which provides sufficient tomographic information thanks to the higher spatial sampling afforded by wide-field illumination at a reduced acquisition time. Moreover, this detachable holder was designed to be compatible with micro MRI system for non-concurrent multimodal imaging. The rotational stage was made from PVC, a non-paramagnetic material. A locking system was fitted onto the FMT system which was capable of rotating and locking the stage at intervals of 90 degrees. A maximum error of ~0.5 degrees was observed between two adjacent views.  Figure 2(b) shows the specimen in the holder on the FMT system. Fiducial markers in form of hollow plastic tubes were incorporated in the holder. These tubes were filled with black ink diluted in water to provide contrast both on the MRI and FMT systems for accurate software registration between the two modalities.

### 2.4. Wide-Field Tomographic Data Set

**Figure 3:**
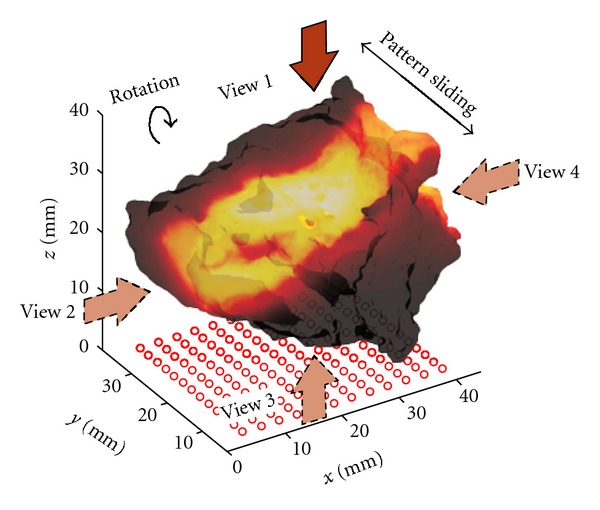
Pattern illumination and detection settings for reconstructing the fluorophore distribution for view 1. The arrows show the projection directions of the patterns. The specimen is rotated along the axis perpendicular to the disk (parallel to the spinal cord).

The spine was illuminated with a homogeneous rectangular pattern covering half the area of the spine. The pattern was translated longitudinally over the spine in the direction of the rotation axis to probe the specimen with 12 pattern locations. This was performed for each of the 4 orthogonal views for a total of 48 patterns acquired. Light was detected in transmittance using 156 fixed detectors (12 × 13 detector array). The detectors were evenly spanned with a separation of 2.48 mm over the spine area. Their position was kept constant above the area of interest during pattern translation. Optimally, this imaging protocol provided 7,488 source-detectors combination. A conceptual depiction of the pattern translation over the spine is provided in Figure 3.

### 2.5. Data Type

Due to the ill-conditioned nature of the optical inverse problem, FMT is known to suffer from low resolution and dilution of contrast and to be sensitive to noise. Among the parameters impacting FMT performances, choice of an optimal data set is critical. CW is the most wide-spread data type employed in FMT due to its simplicity to implement and low cost instrumental components. However, the CW data type suffers from two important disadvantages. First, CW techniques cannot image fluorescence lifetime, an intrinsic fluorophore characteristic that permits one to perform *in vivo* fluorescence multiplexing or allow one to monitor physiological events. Second, due to the limited information content present in the CW data set, resolution is entirely dependent on a tissue's optical properties and geometry; it cannot otherwise be optimized. These caveats can be overcome by employing time-dependent data types.

Recently, several studies have investigated the potential of performing FMT directly using Time Domain (TD) derived data types, such as moments, or time gates [22, 23]. Especially, it is well established that resolution of the optical reconstructions can be dramatically improved by using the rising portion of the TPSFs (termed early-gates) in FMT settings [21, 24] and even in full-field imaging [25]. Combining early- and late-gates provides both increased resolution and quantification in FMT imaging [26]. Herein, in this study, we simulated and/or experimentally collected time-gated data type. To benefit from the optimal time resolution achievable with our system with temporal stability, time gates of 300 ps were employed at 40 ps intervals (120 gates over a 4.8 ns time window). This was performed both for the synthetic measurements and experimental data. For the experimental study, the total acquisition time per view (12 pattern positions) was <5 min for emission and <90 sec for excitation.

### 2.6. Synthetic Model Based on Real Specimen

To investigate the performances of the approach designed herein, we created a synthetic model of the spine derived from a high-resolution anatomical image (MRI) of an experimental specimen. This synthetic model is used to conduct *in silico* studies to evaluate the impact of parameters, such as number of views, optical properties, and time dependent data sets on FMT performances for this specific application. 

#### 2.6.1. MRI of the *Ex-Vivo* Spine

**Figure 4:**
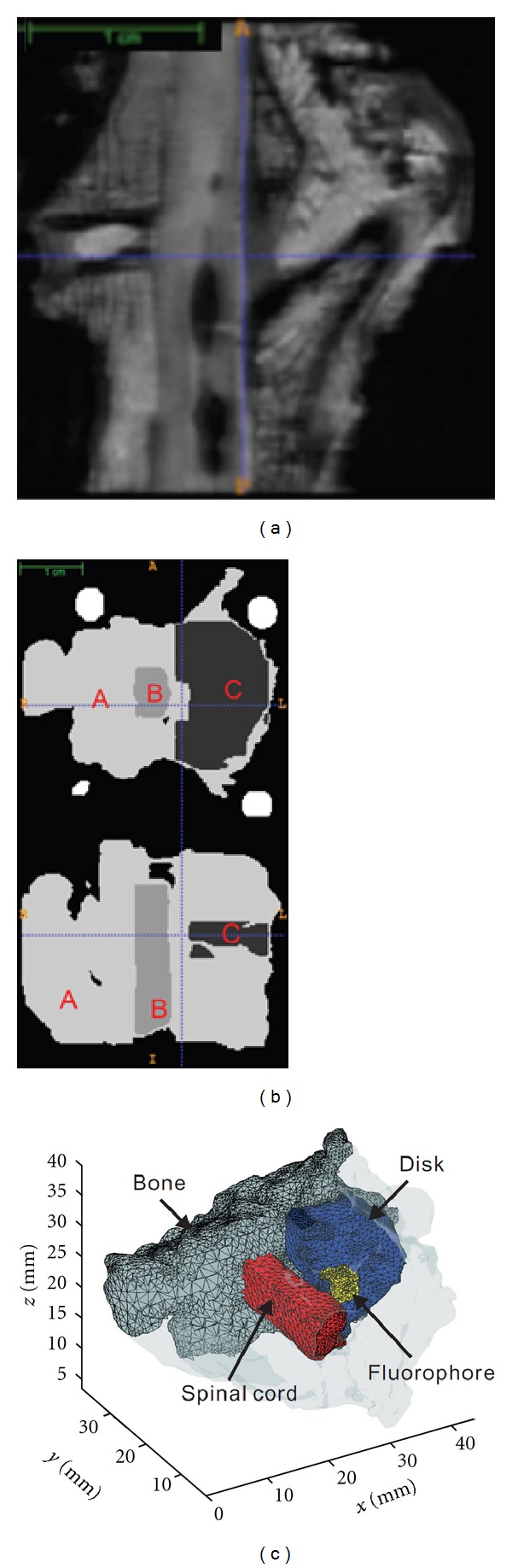
(a) Typical MRI axial slice of the *ex vivo* dog spine; (b) Segmented spine model (A: bone; B: intervertebral disc; C: spinal cord); (c) 3D meshes associated with the three regions (and simulated fluorophore distribution).

The MRI of the spine was carried out using a Bruker Pharmascan 16 cm 7T Horizontal MRI/MRS system. To keep the consistency between MRI and optical imaging sessions, the spine was first loaded into the multimodal rotational stage. This stage was then placed inside the specially manufactured plastic tube. *Ex vivo* MRI requires tissues to be immersed in a suitable medium to prevent sample dehydration and the generation of susceptibility artefacts arising from tissue-air interfaces. Perfluoropolyethers (PFPE) have been used in a number of studies as a wetting and embedding agent for *ex vivo* MRI of biological tissues. The use of PFPE is near ideal having few 1H nuclei and is nontoxic and noninflammable. In this study, we employed Fomblin (Ausimont, Thorofare, NJ). The immersion of the spine in fomblin allowed avoiding sample dehydration during MRI imaging sessions without interfering with MRI contrast. For optical imaging sessions, the tube and fomblin were removed to perform non-contact, matching liquid free, FMT of the spine. The MRI of the spine was performed for 3D high-resolution anatomical information (Echo time = 11.55 ms; repetition time = 2,000 ms; rare factor = 8; field of view = 35 mm × 35 mm × 35 mm; spatial resolution = 0.206 mm/pixel × 0.206 mm/pixel × 0.206 mm/pixel). The total number of slices acquired was 175. The overall time required to complete the MRI imaging procedure was approximately 2 hours. A typical example of MRI images of the spine is provided in Figure 4(a).

#### 2.6.2. 3D Anatomical Segmentation

3D anatomical MRI data of the spine were segmented into three regions: bone, spinal cord, and the intervertebral disc. These three regions were considered to be the main optical regions of interest with differing optical properties for this application. Segmentation was performed using the active contours method using ITK-SNAP.  Figure 4(b) provides the labeled segmented volume of the intervertebral disk within the whole MRI anatomical data (2D representation in the figure for display, but 3D segmentation is obtained). The outcome of the segmentation process is a full 3D rendering of the specimen with 3 labeled regions that are continuous, contiguous, but non-overlapping. However, the segmentation process generates a voxel-based 3D model with high resolution (voxel of 0.24 × 0.24 × 0.24 mm^3^) that cannot be used in FMT due to the associated huge size of the inverse problem. To be able to perform computationally efficient FMT while retaining an accurate boundary, a 3D mesh was created from the 3D voxel segmented data.

#### 2.6.3. 3D Labeled Mesh Generation

Voxel-based mesh generation is commonly done in various computational fields. However, generating 3-D meshes, based on anatomical medical images is still a challenging task, especially in the case of complex boundary conditions. Herein, we employed MeshSim (Simmetrix Inc., NY, USA) to generate 3-D meshes of the spine based on the MRI voxel-based 3D model. MeshSim takes voxel data as input and creates surface or volume meshes. The meshing process is fully automated requiring no user interaction. For this study, MeshSim was modified to be able to generate labeled meshes. This implementation allowed extracting a particular region from the full 3D mesh using the 3D label information obtained from segmentation of the spine in 3 regions.  Figure 4(c) shows the mesh generated using MeshSim for each of the labeled region (note that the overall mesh is contiguous). The full generated 3D mesh contains 469,415 elements, with a denser tessellation around the external and internal boundaries. This number was chosen as a golden mean between processing time and resolution based on our experience [27]. For fluorescent simulations, a 27 mm^3^ fluorescence inclusion (3 mm × 3 mm × 3 mm) was simulated within the disk. The fluorescent object had quantum yield of 0.1 and lifetime *τ* = 0.5 ns. The location of the fluorophore in the intervertebral disk is provided in Figure 4(c).

### 2.7. Monte Carlo Based Optical Reconstructions

The selection of appropriate mathematical models that describe photon propagation in tissue is essential for accurate quantitative imaging. The most common model employed is the diffusion approximation, which is an approximation of the radiation transport equation. However, it is not a suitable model when broad span of optical properties, void regions, or small geometries are encountered and short pulse propagation is considered [28–30]. These conditions are encountered in the case of *ex vivo* spine imaging. Herein, we employed a newly developed mesh-based Monte Carlo code to compute accurately the forward model in this complex sample [27]. The forward model was employed to generate accurate synthetic data for *in silico* investigation, but also to create the Jacobians to perform the optical inverse problem. Both Continuous Wave (CW) and Time-Gated (TG) forward model were used. CW simulations were carried out using 10^7^ photons, whereas the TG simulations were performed with 10^8^ photons. In both cases, wide-field patterned sources replicating the experimental ones (spatial distribution and temporal characteristics) were employed. The Jacobians were computed with the time forward-adjoint method for computational efficiency [31].

To compute the forward model, both homogenous and heterogeneous optical properties (3 regions) were considered. The optical properties employed were derived from the literature and summarized in Table 1. For the homogeneous case, the optical properties of the bone were employed. Note that the homogenous forward model was employed only to compute homogenous Jacobians. In all the work herein, the synthetic measurements employed were based on a heterogeneous forward model.

### 2.8. Inverse Problem

**Figure 5:**
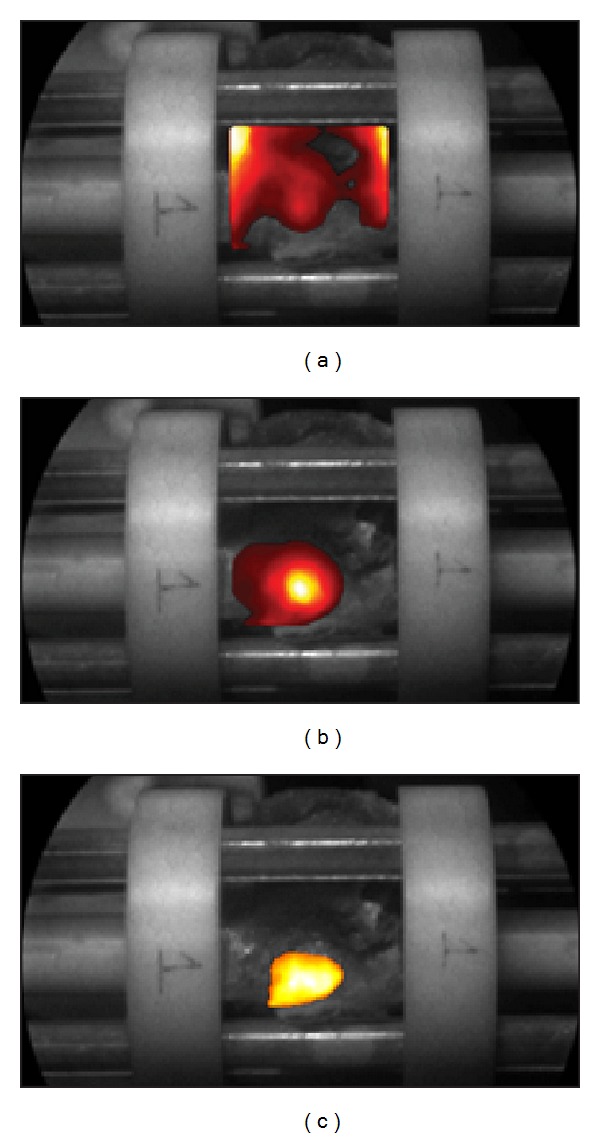
Examples of typical experimental (a) excitation measurement, (b) emission measurements, and (c) Born normalized data. The images were thresholded based on photon counts for visualization (200 counts).

Besides selecting the optimal data set for reconstructions and employing dense data sets, many strategies have been devised to provide more robust reconstruction. Among these, one scheme is paramount towards quantitative imaging. It consists of utilizing normalized data by dividing the experimental fluorescence data by the excitation data [32]. A classical mathematical expression of this formulation is:

(1) UBn(rs,rd,t)=[ΘU0(rs,rd)∫Qd3r∫0tdt′∫0t′dt′′Gx(rs,r,t′−t′′) ·Gm(r,rd,t′′)·η(r)·e−(t−t′)/τ],

where *U*_*B*_^*n*^(*r*_*s*_, *r*_*d*_, *t*) is the normalized fluorescence Born photon density at position *r*_*d*_ from a source located at *r*_*s*_; *U*_0_(*r*_*s*_, *r*_*d*_) is the transmitted excitation homogeneous photon density (note that it is the integrated excitation photon density over time); *G* is the propagator; *η*(*r*) is the product of the fluorochrome absorption coefficient, fluorescent quantum yield, and quenching factor; *τ* is the fluorochrome lifetime; and Θ incorporates unknown constants associated with wavelength-dependent gains and attenuations that can be measured once for every imaging system. Finally, for the fluorescence reconstruction, the volume of interest is segmented into mesh elements and (1) is discretized on the assumed mesh. The normalization of the fluorescence measurements by the excitation measurements allows reducing the impact of the heterogeneous background on the localization and quantification of the fluorochrome [33]. Moreover, this scheme allows for less stringent calibration protocols as instrument dependent parameters are canceled out in the normalized formulation. An example of experimental excitation, emission, and Born normalized data is provided in Figure 5.

**Figure 6:**
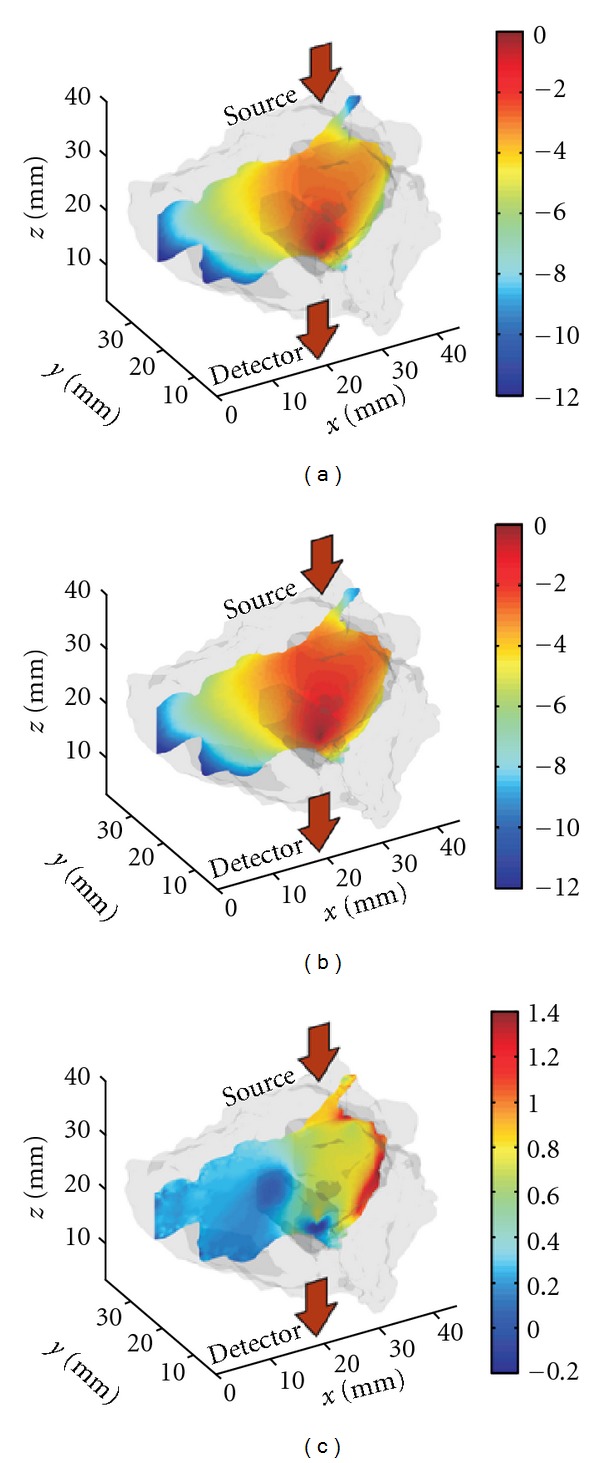
It shows an example of Jacobians for CW simulations: (a) homogeneous Jacobian, (b) heterogeneous Jacobian for the same optode pair, and (c) the difference between the Jacobians. The illumination is on the left-hand side of the spine and the detector located on the right-hand side (at the solid red position). Both Jacobians are normalized to their respective maximum.

However, such approaches may not reduce completely the effect of background optical properties on the fluorescence reconstructions. Hence, we computed Jacobians based on both homogenous and heterogeneous optical properties within the Born normalized formulation. An example of Jacobians computed in CW for homogeneous and heterogeneous model is provided in Figure 6. 

In the case of time gated reconstruction, the rising gates at 25% of the peak photon counts were first used for improving resolution, then this result was set as the initial guess of a late-gate reconstruction (50% decaying) for quantification accuracy. All reconstructions herein were obtained using a least square method stopping after 100 iterations. No soft or hard priors were included in the optical inverse problem. 

## 3. Results and Discussion

### 3.1. *In Silico* Evaluation

**Figure 7:**
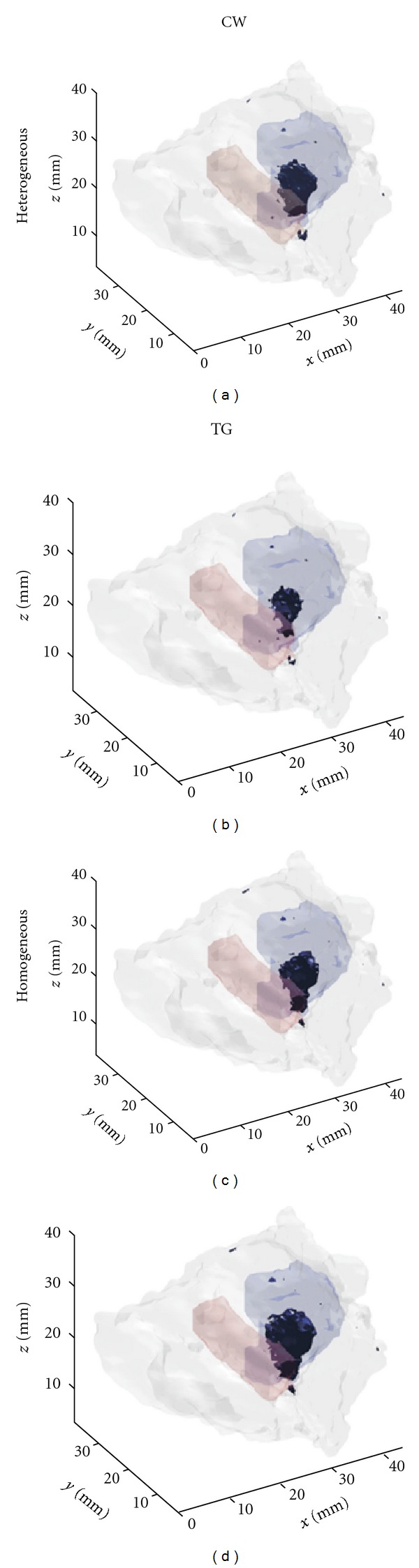
Reconstructions based on 4 views for (a) and (c) CW data type and (b) and (d) TG data type. The 50% isovolume is displayed and the disk/spinal cord is highlighted with light-grey shading.

The aim of the *in silico* study was two-fold. Firstly, the simulations were conducted to evaluate the performances of wide-field time gated FMT to accurately image a fluorophore localized in the intervertebral disk. Parameters such as data type, number of views, and impact of optical properties were investigated. The reconstructions fidelity was assessed in terms of localization and quantification. Second, these simulations were employed to guide and refine the experimental imaging protocol.  Figure 7 provides the 50% iso volume rendering of the reconstructed fluorophore in the case of 4-view reconstructions for all cases investigated. This visualization demonstrates the size of the reconstructed object using different reconstruction settings and clearly shows the improved fidelity in reconstructions when using time-gated heterogeneous Jacobians. It is worth noting that in all cases studied, the *z* dimension of the isovolume is larger than the *x* and *y* ones, implying a loss of resolution for *z* axis. This is potentially resulting from the illumination-detection settings due to the sliding direction (along *y*) and the detection views (missing *x*-*y* detectors).

To evaluate the localization accuracy in recovering the simulated fluorescent inclusion, the centroid position of the 50 percent isovolume of the reconstructed object and the actual object were calculated. *X*, *Y*, and *Z* coordinates of corresponding centroids were compared and Euclidian distance errors (localization error) were calculated based on these coordinates in millimeters. The *X*, *Y*, and *Z* axes were defined with respect to the spine. These axes were kept constant irrespective of the view.

The simulation results were also evaluated based on their quantification performance. The evaluation was done through comparison between the expected value and the observed fluorescence value for the 50 percent isovolume. This comparison was made by calculating relative errors between the observed value and the expected value with respect to the expected value, as provided by:

(2) $$\begin{array}{c} \text{Relative}\;\text{Error}=\left|\frac{\text{Observed}-\text{Expected}}{\text{Expected}}\right|\times 100. \end{array}$$

The volume of the reconstructed object was also estimated based on the 50 percent isovolume threshold employed above.

Table 2 summarizes the localization error, volume reconstructed, and quantification accuracy in the case of CW and TG data type for each independent view (90 degrees apart) and combining 4 views, using both homogeneous and heterogeneous Jacobians.

Overall, in all cases investigated herein, the fluorophore was reconstructed in the disk with good localization accuracy. The fluorophore was always localized with less than 2 mm error and with only 0.5 mm inaccuracy in the best case scenario (TG-heterogenous). As expected, multiple views gave better localization compared to single views. When the views were combined, the localization errors were half of the smallest errors achieved for individual views in case of the heterogeneous model. In case of the homogeneous model, although an overall significant reduction was observed, the multi-view localization error was matching the minimum error of the individual views. Also, as expected, time-gated FMT outperformed CW reconstructions in terms of localization. However, the gain in accuracy in using TG over CW is not commensurate with the gain of using multiple views, suggesting that localization accuracy is more affected by the geometrical sampling strategy than the data type employed. Based on multiple views, accurate localization of the fluorophore can be achieved within the order of the mesh discretization scale (0.3 mm). The localization errors reported in Table 2 fall below 2% of the actual dimension of spine along its smallest dimension for the best reconstruction scheme (~5% for the worse case). 

In terms of quantification, the different strategies investigated herein exhibited similar trends than the localization parameter. The best quantification was achieved with TG data set and heterogeneous Jacobian (89.36%) while the worst case was achieved in using CW data and homogenous Jacobian (59.97%). Combining multiple views improved quantification. The main difference between localization and quantification trends lies in the data type. TG data do significantly better than CW data overall. To better understand this aspect, we provided also the volume of the object reconstructed (as estimated with the 50% iso-volume). As seen, the volume of the fluorescent heterogeneity is significantly smaller when using TG and multiple views (expected 27 mm^3^). The contrast retrieved is then more focal and hence, better quantified. As described previously, the reconstruction scheme employed herein capitalizes on the early gate data to improve resolution. The incorporation of this data type reduced by half the volume of the object reconstructed. Even in the case of homogeneous Jacobian, the incorporation of the early gate led to reliable reconstructions. This indicates that multiple view and TG data type can be used for accurate FMT of the sample without the necessity to generate a heterogeneous model. This eliminates the necessity of acquiring high-resolution anatomical images which can be a tedious and costly step. Note however, that high-resolution a-priori information could be employed in the optical inverse problem to increase the robustness and improve the resolution of fluorescence reconstructions [34]. Moreover, in this study, we limited ourselves to the acquisition of 4 views to allow for 3D imaging of the full specimen with short experimental acquisition time. It is expected that increasing the number of angular view in the acquisition will lead to improved resolution [35]. For instance, Niedre and Ntziachristos demonstrated improved resolution using early time gates and dense angular sampling over continuous wave data type [36], though studies based on 360 degrees optical tomography in FMT are limited to 2D imaging due to prohibitive acquisition times [37, 38]. Wide-field strategy allows significantly reducing the acquisition time by inherently sensing large 3D volume and providing measurements with higher SNR. We expect that by limiting the number of gates acquired (120 in this study) to a few, we can increase angular sampling for improved resolution. The optimal combination of gates and angular projections will be the focus of subsequent studies.

### 3.2. Experimental Reconstructions

**Figure 8:**
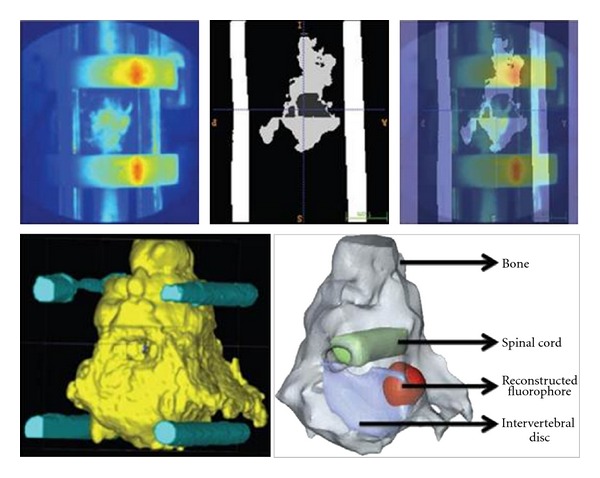
3D visualization of the reconstructed fluorophore. The disc (blue region) and the spinal cord (green region) are provided but obtained from the MRI segmented model.

An experimental validation of the methodology was performed using the specimen employed to create the synthetic measurements. The same settings as the simulations were used experimentally (12 patterns-156 detectors/view, 300 ps gate witdh, etc.). Both excitation and emission measurements were collected sequentially for three views (exclusion of view 3 due to poor SNR).  Figure 2(c)-2(d) shows the emission measurements for two views. The positioning of the spine was such that the area of interest was exactly below and within the field of view of the camera (detector). The experimental data were registered with the *in silico* model using the optical and MRI fiducials. The model was rescaled through linear scaling and resampled to match the experimental images. Registration was performed by pixel-to-pixel matching. This registration process allowed employing the synthetic spine model for the experimental reconstructions.  Figure 8 shows the reconstructed fluorophore superimposed on the 3D spine model. The location on the reconstructed fluorophore distribution exactly matched the location of the injection site of ICG.

## 4. Conclusion

In this study, we have investigated the potential of wide-field FMT to image nondestructively *ex vivo* sample, in this specific case, the dog spine. This computational and experimental study demonstrated the ability of wide-field FMT to accurately retrieve the biodistribution of a fluorophore localized in the intervertebral disk with high accuracy, both in terms of localization and quantification. To model accurately such complex model, a mesh-based Monte Carlo reconstruction approach was employed and for optimal imaging fidelity, time-gated data sets were employed. As expected the TG data sets, when combining early and late gates, outperformed significantly the CW data sets in terms of localization and quantification and the use of multiview imaging protocols led to more accurate localization. Additionally, the incorporation of the heterogeneous nature of the tissue in the model to compute the Jacobians led to improved imaging performances. However, these improvements could be considered minor compared to the added difficulty to obtain such a-priori information. Indeed, such a priori information requires the use of high-resolution anatomical imaging modality, which increases significantly costs and imaging time protocols. The entire experimental process for this investigative study took approximately <30 minutes and can be drastically reduced by acquiring only the necessary information for FMT (for instances, 6 gates versus 120 gates in this study). In conclusion, this successful experimental imaging study provides a proof of concept of the feasibility of quantitatively imaging complex *ex vivo* samples nondestructively and with short acquisition times. This work is the first step towards employing optical molecular imaging of the spine to detect and characterize disc degeneration based on targeted fluorescent probes.

## Figures and Tables

**Table 1:** Optical properties assigned to the different segmented region of the spine [8, 9].

	*μ*_a_ (cm^−1^)	*μ*_s_ (cm^−1^)
Bone	0.04	12
Spinal cord	0.121	8.9
Disc	0.008	5

**Table 2:** Localization and quantification comparison for reconstructions in the spine (expected volume = 27 mm^3^; expected quantification = 100%).

		Localiz. error (mm)		Volume (mm^3^)		Quantif. (%)	
	View	CW	TG	CW	TG	CW	TG
	1	1.01	0.91	85.21	79.77	48.12	52.12
	2	1.04	1.02	63.21	53.75	72.35	73.21
Hete.	3	1.26	1.10	105.22	89.67	56.51	49.77
	4	1.54	1.47	120.56	119.88	38.75	45.77
	Comb.	0.78	0.50	89.75	37.53	73.43	89.36

	1	1.62	1.08	102.33	85.54	32.54	44.84
	2	1.25	1.79	75.65	67.64	51.15	57.44
Homo.	3	1.47	1.22	119.42	92.75	45.86	39.76
	4	1.71	1.60	137.63	112.38	34.91	38.77
	Comb.	1.28	1.21	93.65	54.75	59.97	72.64
